# Digital communication activities and EFL learners' willingness to communicate and engagement: Exploring the intermediate language learners’ perceptions

**DOI:** 10.1016/j.heliyon.2024.e25213

**Published:** 2024-01-29

**Authors:** Ruyu Han, Goudarz Alibakhshi, Lu Lu, Akram Labbafi

**Affiliations:** a Foreign Language Teaching Department, Shanxi Finance and Taxation College, Taiyuan, China; b Allameh Tabataba'i University, Iran; c School of Foreign Languages, Wuhan Qingchuan University, Wuhan, China; d English Language Teaching, Islamic Azad University, Maraghe Branch, Maraghe, Iran

**Keywords:** Digital communication activities, Chinese EFL learners, WTC in the classroom, Learner engagement

## Abstract

EFL/ESL teachers have used digital communication activities to teach language skills. However, the effect of digital communication activities on EFL learners' Willingness to Communicate (WTC) in classrooms and learner engagement has yet to be well investigated. This study examined the influence of digital communication activities on the engagement and willingness to communicate of intermediate English as a Foreign Language (EFL) learners. It also assessed the potential advantages of integrating digital communication into language learning contexts. A mixed-methods approach involving pretest-posttest comparisons and qualitative interviews was employed. In the quantitative phase, four intact classes of 80 intermediate Chinese EFL learners were recruited and assigned to control and experimental groups. They attempted the scales (WTC and engagement) before and after treatment. However, 20 EFL learners exposed to digital communication activities were interviewed. The research revealed notable enhancements in affective, cognitive, and behavioral engagement among the experimental group. Moreover, a substantial positive effect on EFL learners' willingness to communicate was observed, particularly in speaking, writing, reading, and comprehension activities. Findings have practical implications for EFL teachers and learners to use digital communication activities to enhance the learners' WTC in the classroom and different aspects of engagement.

## 1 Introduction

Digital communication refers to exchanging information through electronic devices like computers, mobile phones, and the Internet. This type of communication has become increasingly popular as technology has made it easier to share information and connect with others [1]. As a result, digital communication has transformed the traditional classroom setting, creating new opportunities for learning and personal development, according to Ng [2]. Distance learning, for example, has become more accessible, as learners can enroll in courses of their choice without being constrained by time and place. Online learning has also expanded the reach of education, attracted new target groups, and made learning more interactive. Peer tutoring, for instance, can be conducted online, discussions can take place on various platforms, and learners can access new information online. Digital communication has also expanded the scope of learning beyond academic excellence, enabling learners to acquire life skills and practical knowledge. Electronic books and educative videos, for example, are readily available online. The use of technology in education has become increasingly important, as possessing technology skills is essential in the 21st century [3]. Educational systems are now being evaluated based on the level of technology integration, as digital communication has been shown to improve performance and motivation among learners. Advanced visual technology in teaching has reduced the time taken to convey information and enhanced learners' ability to retain it.

Digital communication has significantly impacted the role of teachers in the classroom [4,5]. With the shift towards digital technology, teachers are no longer seen as the sole source of knowledge but as facilitators and guides to help students navigate the vast amount of online information [6]. Teachers are expected to be proficient in the use of digital tools and to incorporate them into their teaching practices to enhance teaching and learning [7]. This includes using digital resources to supplement traditional teaching materials, creating online learning environments, and utilizing collaborative tools to engage students in learning. Additionally, teachers are expected to adopt a more flexible and personalized approach to teaching, where they can tailor their instruction to meet the diverse needs of students [5]. This requires teachers to be more adaptable and innovative in their teaching strategies [7], as well as to be able to provide constructive feedback to students in a digital environment [8]. Overall, the use of digital communication has transformed the role of teachers to one that is more collaborative, flexible, and technology-savvy [[1], [2], [3], [4], [5]].

Digital communication activities affect the learners' affective, cognitive, and educational variables. One of the affective variables that affect language learners' language progress is language learners' engagement in classroom activities. Learner engagement is the commitment of a student's cognitive and emotional resources to accomplish a learning task. It has been consistently associated with significant educational outcomes, including academic achievement, persistence, satisfaction, and a sense of community [8,9]. These correlations have led scholars to characterize learner engagement as “an educational bottom line” and “the holy grail of learning [10]. Despite these associations, many students must be more actively engaged in their education, resulting in high attrition rates and diminished interest, motivation, and academic outcomes. Facilitating students' active engagement in the learning process constitutes a significant subject of investigation within the realm of instructional technology research [8,9]. Notably, most of the assessed scholarly works needed a distinctly articulated definitional framework concerning the construct of engagement [8,9]. Although a universally standardized conceptualization of student engagement remains elusive, scholars concur that engagement constitutes a multifaceted and intricate phenomenon encompassing discrete sub-constructs, as corroborated by an array of indicative metrics. Emotional and cognitive engagement emerge as pervasive and fundamental sub-constructs, garnering particular emphasis within blended learning context (10). Emotional engagement denotes the affective reactions exhibited by students when confronted with various facets of the learning process, encompassing learning tasks, contextual elements, peer interactions, and instructor dynamics, thus encompassing sentiments such as affiliation, curiosity, joy, and the like, thereby encompassing both affirmative and negative affective responses.

Meanwhile, cognitive engagement encapsulates students' cognitive investment in tackling academic tasks to fathom and master subject matter. This facet encompasses metacognitive strategies, a penchant for embracing challenges, and adeptness in self-regulatory proficiencies [11,12]. Notably, emotional and cognitive engagement continue to influence academic attainment significantly. This study focuses mainly on the language learners' affective, cognitive, and behavioral engagement.

Another variable that can be affected by digital communication activities is language learners' willingness to communicate (WTC). A learner's willingness to participate in communicative activities is a critical factor in determining their engagement in communication. This willingness is referred to as willingness to communicate (WTC) in L2 research. It is the readiness to enter into discourse at a particular time with a specific person or persons using an L2 [[13], [14], [15], [16], [17], [18]]. This definition reflects the dual nature of L2 WTC as both a trait and a state construct [19,20]. As such, research on L2 WTC has investigated its antecedents as either a trait or a state. Studies that view L2 WTC as fluctuating have explored the relationship between situational factors, such as activity types, topics, interlocutors, classroom climate, class sizes, teaching styles, and L2 WTC [21,22].

The connection between digital communication activities and the willingness to communicate (WTC) is a dynamic and multifaceted relationship that unfolds within the evolving landscape of contemporary communication. Digital platforms offer various channels, including social media, instant messaging, video conferencing, and collaborative online spaces, influencing how individuals express themselves in a second language. One key aspect lies in the motivation fostered by digital interactions. The accessibility and immediacy of communication in the digital realm can enhance learners' motivation to engage in language exchange as they perceive a real-world application for their language skills. Additionally, the digital environment provides a unique space for learners to build self-efficacy. Navigating various digital communication tools and adapting to different online communication norms can contribute to learners' confidence in using the second language [[15], [16], [17]]. However, challenges such as digital communication-induced anxiety may also emerge, impacting learners' WTC. The pressure to communicate effectively in an online setting, fear of misinterpretation, or apprehension about the permanence of digital interactions can influence learners' willingness to participate actively. Understanding these nuances is crucial for educators and researchers seeking to unravel the complex interplay between digital communication activities and the desire to communicate in second language acquisition [[18], [21], [22]]. On the other hand, some research has considered L2 WTC as a constant individual trait and examined its antecedents, such as gender, age, cultural background, anxiety, motivation, and personality [[19], [20], [23], [24], [25], [26], [27], [28], [29], [30], [31], [32], [33]].

The existing literature on digital communication activities and English as a Foreign Language (EFL) learners' willingness to communicate (WTC) and engagement primarily focuses on broad aspects, leaving a notable gap in understanding the nuanced perceptions of intermediate language learners. While there is ample research on the general influence of digital communication on language acquisition and communication apprehension, there needs to be more studies specifically delving into how intermediate EFL learners perceive and experience digital communication activities and their willingness to communicate. This research aims to address this gap by exploring the intricate dynamics and perceptions of intermediate EFL learners regarding digital communication, shedding light on the factors that either facilitate or hinder their WTC and overall engagement in language learning through digital platforms.

### 1.1 Research questions

In line with the objectives of the study, the following research questions are stated:

1. Do digital communication activities have a statistically significant effect on EFL learners' engagement?
2. Do digital communication activities have a statistically significant effect on EFL learners' Willingness to communicate (WTC)?
3. What are the intermediate language learners' perceptions of digital communication activities?

## 2 Review of literature

### 2.1 Theoretical background

The study is mainly based on two leading theories: Sociocultural and cultural theory and cognitive theory of learning; each is explained as follows.

### 2.2 Sociocultural and cultural theory

The sociocultural theory posits a significant connection between an individual's psychology and the cultural and institutional context in which they are situated [19]. As Ahmed [20] articulates, culture encompasses inherited beliefs and practices that exert a substantial influence on the course of our lives. Central to this theory is the role of social interactions and cultural engagements in shaping psychological development. It underscores that development is not solely an internal process but is profoundly impacted by external social interactions. The surroundings in which individuals find themselves play a pivotal role in shaping behavior and learning. In this view, language mirrors and communicates the very fabric of culture.

ESL learners enhance their language proficiency through interactions with native speakers and under mentorship. Digital communication platforms immerse learners in sociocultural environments, with the language used on these platforms significantly affecting language improvement. The depth of word meanings is gleaned through communication [20,26]. Peer interactions are equally crucial, guided by instruction as emphasized by the theory [19].

### 2.3 Cognitive Theory of Learning

The Cognitive Theory of Learning elucidates how individuals process and retain information to facilitate effective learning [28]. This theory accentuates the importance of comprehension in learning, fostering creativity. Essential language skills within this framework encompass listening, speaking, reading, and writing. ESL learners must tap into diverse language resources to maintain motivation and practice. According to the theory, language learning necessitates cognitive effort, with outcomes contingent on input and interaction [29]. Repetition asserts it enhances learning quality by bolstering memory retention. Learners who repeatedly engage in repetitive tasks or revisit content exhibit higher language proficiency and information absorption. Javed [30] further posits that effective language skill development necessitates teaching, thorough explanations, and practice.

### 2.4 Digital communication activities and ESL/EFL learning

Technology has played a pivotal role in the evolution of learning media, facilitating instructional communication to enhance student learning and teaching practices [31]. Technology offers platforms for theoretical, informational, and experiential content. A study by Viberg and Gronlund [32] underscored the alignment between learning methodologies and technological integration, advocating for incorporating technology to harmonize theory and practice. The research contends that curriculum design should recognize learners' frequent use of phones and laptops for self-directed learning activities, prompting the need for tools tailored to these learning practices. Consequently, language curricula should adapt to individualized learning by integrating technology to accommodate students' routine usage. These findings are corroborated by Mellati et al. [31], who assert that learners can enhance vocabulary and other language skills by utilizing social networks as platforms for practice. This study focusing on technology's role in language learning highlighted learners' preference for individualized learning at their own pace, indicating a reluctance to invest excessive time in learning endeavors. Learners tend to refrain from curtailing their mobile device usage for study purposes. The internet, identified as an interactive and educational milieu, provides an audience for creative expressions, with electronic devices fostering innovative and varied learning environments [31,32].

Online learning platforms enable the formation of learning communities, empowering learners to construct and disseminate knowledge [20,33]. Sari & Magana [34] emphasize that language proficiency encompasses comprehending others and responding effectively, with media as a conducive platform for developing this skill. Sockett's [35] endorsement of these findings underscores that practicing ESL skills beyond the classroom positively influences learners' language proficiency, instilling confidence. Furthermore, the informal nature of online social media environments could render ESL learners unaware of the language proficiency gains resulting from online activities. Lai et al. [36] expand these findings by asserting that learners who engage in extracurricular practice and interaction exhibit elevated language proficiency, benefiting from the diverse environment. Digital communication augments English language knowledge and vocabulary and fosters a positive attitude and motivation toward language learning [36,37]. Online platforms create a comfortable space that boosts learners' willingness to converse in English. Consequently, diminished teacher dominance empowers learners to express themselves more effectively and refine their communication skills.

Social networking platforms like Facebook have emerged as prominent channels for English communication, facilitating idea exchange and deliberative discussions [20]. Dweika [33] investigated the impact of a dedicated Facebook group for English as a Second Language (ESL) learners on language proficiency. Students engaged in this group showed significant improvements in their English language skills, prompting recommendations for educators to incorporate Facebook to enhance connectivity and resource sharing. Ahmed [20] conducted a similar study, demonstrating that students participating in English-focused dialogues on Facebook displayed improved writing and grammatical abilities.

As another social network, YouTube serves as a platform for ESL learners to engage with diverse English content, refining language skills across various domains [34]. Raj et al. [38] found that using YouTube for language learning enhances vocabulary and eloquence. Learners can also improve their creative faculties by participating in discussions and recording videos. Sockett [35] highlighted how music content and YouTube lyrics improve pronunciation and writing skills. Holmberg (2019) substantiated these findings, showcasing improved vocabulary and communication skills among YouTube-engaged learners.

Moreover, video conferencing potentiates speech confidence and global competency [35]39. Lee [39] underscored the value of video conferencing in enhancing oral proficiency and enabling online interactions with native speakers. Alamrani [40] evaluated the significance of digital communication, indicating its positive influence on ESL learning, dependent on technical access and issue resolution.

## 3 Willingness to communicate

The concept of Willingness to Communicate (WTC) pertains to individuals' volition to partake in verbal exchanges with designated individuals or groups, utilizing a secondary language (L2) [41]. It can also be construed as an enduring propensity for discourse when afforded the liberty of choice [42]. Kurk [43] extends this notion by asserting that WTC mirrors a learner's cognitive deliberation in harnessing the target language for communicative purposes. Within this context, MacIntyre and Vincze [44] posit that WTC is the paramount objective in acquiring foreign languages, given its potential to stimulate genuine communicative conduct and enhance proficiency in the L2. As delineated by Öz et al. [45], the comprehensive construct of WTC encompasses affective, socio-psychological, linguistic, and communicative parameters. This construct assumes the role of elucidating and prognosticating language learners' communicative tendencies within the L2 domain. MacIntyre et al.'s [41] theoretical framework propound a trichotomous analysis of WTC, clarifying its examination through trait-oriented, dynamic, and contextual lenses [46].

The psychological facet of WTC is intimately intertwined with foreign language anxiety, self-assurance, and motivation [47]. Conversely, the dynamic and contextual dimensions of WTC are entangled with the socio-environmental and situational constituents of the learning process, encompassing factors such as conversational partners [48], discourse topics [49], educators [50], and collaborative peers [51].

Recent literature has prominently advanced the notion that Willingness to Communicate (WTC) is best apprehended as a dual-faceted construct, amalgamating the learner's enduring traits and situational dispositions [[52], [53]]. This bifocal perspective underscores WTC's derivation from stable learner traits, such as age, gender, and personality [54]. Concurrently, it acknowledges its susceptibility to fluctuation contingent upon situational cues, encompassing interlocutors, pedagogical methodologies, and thematic contexts [55]. Given its intimate connection with learners' inclination to actively seek communicative opportunities and engage in interactive exchanges [56], WTC is pivotal in language acquisition.

A pervasive postulate within the second language (L2) domain posits WTC as a pivotal determinant of L2 communicative conduct, thereby conducing L2 proficiency [57]. A cluster of studies has probed into WTC's capacity to forecast L2 communicative patterns, with findings suggesting a positive correlation between heightened WTC and augmented L2 engagement [56,57]. Moreover, inquiries have scrutinized the nexus between WTC and L2 competence, revealing a constructive association [58]. More recently, scholarly investigations have illuminated that L2 performance is contingent upon learners' WTC, thus transcending mere communicative behaviors.

### 3.1 Student engagement

Emotional engagement denotes the affirmative and adverse reactions students exhibit towards peers, educators, educational institutions, and learning outcomes. Conversely, cognitive engagement is characterized by students' intellectual investment in and comprehension of subject matter, encompassing meticulous contemplation and a willingness to invest substantial effort in comprehending intricate concepts and mastering arduous skills [59]. The ramifications of academic engagement are manifold and enduring, encompassing endeavors such as pursuing advanced education, sustaining consistent learning habits, enhancing vocational opportunities, nurturing constructive self-conception and well-being, and mitigating symptoms of depression [60]. Consequently, dynamic involvement in academic pursuits engenders positive outcomes that transcend the confines of educational contexts. Furthermore, intellectual engagement evinces a robust nexus with academic motivation and performance, as students who actively participate in scholarly endeavors are inclined to accord higher evaluations to their studies, attain elevated scores, and evince diminished levels of academic disengagement and evasion [61]. Recently, engagement has garnered substantive consideration as a pivotal determinant of literary triumph. It is posited that positive emotions indirectly influence academic outcomes through motivational mechanisms, prominently exemplified by engagement [[42], [62], [63], [64], [65]]. In this paradigm, engagement is a pivotal driver of educational aspirations.

Students who manifest keen interest are apt to channel augmented exertions toward academic tasks, culminating in successful task completion and an elevation in academic performance [65]. In professional milieus, engagement is characterized as a mental state characterized by heightened vigor, unwavering dedication, and complete engrossment [[42], [62], [63], [64], [65]]. Vigor underscores heightened cognitive dynamism during work; dedication encapsulates a sense of self-value, enthusiasm, inspiration, pride, and challenge, while engrossment entails complete absorption and gratification in one's undertakings, leading to a swift passage of time. This conceptual framework has been transposed into the academic realm, focusing on students' academic tasks and activities [65]. Engaged students experience heightened vitality, an emotional attachment to their academic pursuits, and an active integration into their scholarly journey [66]. Empirical substantiation bolsters the proposition that engaged university students exhibit enhanced academic performance [65], with practical designs unveiling a positive correlation between engagement and educational attainment [66]. Engagement correlates with elevated academic grades, educational accomplishment, and self-reported learning achievements [[66], [67], [68]]. Succinctly, engagement emerges as a pivotal catalyst for academic success, wherein affirmative emotional states catalyze augmented engagement, ultimately conducing to enhanced academic performance. Engaged students are predisposed to channel escalated effort into their educational undertakings, thus fostering triumphant task execution and elevated scholastic accomplishment. Therefore, educators are urged to cultivate academic engagement by developing a positive pedagogical milieu, nurturing positive affective states, and fostering active participation in academic pursuits.

## 4 Methodology

### 4.1 Research design

The research method employed in this study was a mixed-method approach, which combines both quantitative and qualitative research designs. The choice of a mixed-method design for this study is grounded in the belief that it offers a more comprehensive and valid exploration of the complex relationship between digital communication activities and English as a Foreign Language (EFL) learners' willingness to communicate (WTC) and engagement. Employing both qualitative and quantitative methods enables a more holistic understanding by triangulating diverse data sources. The qualitative component allows for an in-depth exploration of learners' subjective experiences, capturing the nuanced perceptions and attitudes that may not be easily quantifiable. On the other hand, the quantitative component ensures statistical rigor and generalizability, providing numerical data that can reveal patterns and trends across a larger sample. Combining these methods enhances the study's validity by allowing for a more robust validation of findings through cross-verification. By employing a mixed-method approach, this research seeks to capitalize on the strengths of both qualitative and quantitative methodologies, offering a more comprehensive and nuanced exploration of the research questions and ultimately contributing to a richer and more reliable understanding of the topic. Specifically, a pretest/posttest control/experimental group research design was used as the quantitative component of the study. This design involved the administration of pretests and posttests to both a control group and an experimental group to determine the effect of an intervention on a particular outcome. The quantitative data collected from this design were analyzed using statistical methods.

In addition to the quantitative design, the qualitative case research design was used as the qualitative component of the study. Phenomenology is a qualitative research approach that seeks to understand the meaning and essence of lived experiences and perceptions. In this study, phenomenology was used to explore the subjective experiences and perceptions of participants in the experimental group regarding the intervention. The qualitative data from this design were analyzed using thematic analysis to identify common themes and patterns in the participants' responses.

One of the key advantages of using phenomenology is that it allows for a rich and detailed exploration of the participants' experiences and perceptions. It enables researchers to understand the complexities of human experiences and to explore the underlying meanings and essence of those experiences. This can be particularly useful when the research question is complex and multi-faceted, as it allows for a more nuanced understanding of the phenomenon being studied. Another advantage of using qualitative study is that it provides a flexible and open-ended approach to data collection and analysis. This approach is well-suited to exploring subjective experiences and perceptions, as it allows participants to describe their experiences in their own words and allows the researchers to identify common themes and patterns in the data.

### 4.2 Participants

The study involved a total sample size of 80 freshman language learners from Shanxi Finance and Taxation College, Taiyuan, China, distributed across four intact classes. Two classes were randomly assigned to the control condition, while the others were assigned to the experimental condition. Additionally, 20 language learners were selected for the qualitative phase of the study. All participants were native speakers of China and were studying English as a foreign language. Participants were selected based on specific inclusion criteria, such as being a freshman at the University from Shanxi Finance and Taxation College and having Chinese as their first language. The participants for the qualitative phase of the study were chosen based on their willingness to participate in semi-structured interviews and their ability to provide rich and detailed accounts of their experiences with the intervention. Among the participants, 45 were female, and 35 were male freshmen learners of English as a foreign language. The participants' age fell within the range of 19–30 (Mean = 23.5, SD = 3.2).

### 4.3 Instrumentation

This study employed three instruments to collect data: the Willingness to Communicate (WTC) scale, the learner engagement scale, and an interview checklist. The WTC and FLCA scales were used as quantitative measures, while the interview checklist was used as a qualitative measure.

To evaluate the Willingness to Communicate (WTC) among English language learners, we employed an assessment tool derived from MacIntyre et al.'s (2001) framework. This instrument encompassed 27 items, each gauged using a specific rating scale. Specifically, participants were requested to indicate their level of agreement or willingness on a scale comprising five points: “Almost never willing,” “Sometimes willing,” “Willing half of the time,” “Usually willing,” and “Almost always willing.” To examine the internal consistency of the items within this assessment, we utilized Cronbach's alpha coefficient. The reliability analysis results offer insights into the degree to which the items in the evaluation consistently measure the construct of interest. The Cronbach's alpha for each dimension exceeded 0.78, indicating that the scale and dimensions enjoyed high internal consistency.

The second instrument was the Student Engagement Scale, a self-report measure that assesses the extent to which students are engaged in classroom activities. This scale measures three dimensions of engagement: Affective Enjoyment, Cognitive Engagement, and Behavioral Engagement. The total score ranges from 12 to 60, with higher scores indicating higher levels of attention [59]. The interview checklist was used to guide semi-structured interviews with participants in the experimental group. The list consisted of open-ended questions and prompts designed to elicit participants' perceptions and experiences of the intervention. The researchers developed the checklist specifically for this study and reviewed by field experts to ensure its validity. The interview checklist was confirmed by three colleague's experts in qualitative research methods; all questions were relevant to the objective of the qualitative phase of the study.

### 4.4 Procedure

We employed an explanatory sequential mixed methods design to investigate the effectiveness of an intervention designed to increase language learners' willingness to communicate in English and engagement in learning processes. First, the study was conducted through a structured series of steps, ensuring a systematic approach to investigating the connection between digital communication activities, English as a Foreign Language (EFL) learners' willingness to communicate (WTC), and engagement levels. Second, the initial phase involved selecting participants from an intermediate-level EFL population. Participants were chosen based on varying backgrounds and language proficiencies to ensure diversity and representation. After screening and obtaining consent, they were randomly assigned to either the control or experimental group. Third, participants' baseline willingness to communicate and engagement levels were measured through validated pretest instruments before any intervention. These instruments assessed participants' comfort and readiness to communicate in English and their motivation and involvement in learning activities.

Fourth, the experimental group engaged in structured digital communication activities over a 16-week semester. These activities were meticulously designed to leverage various digital platforms such as social media, online discussion boards, and video conferencing to facilitate real-time communication, collaborative discussions, and exposure to authentic language use. Fifth, following the completion of the digital communication activities, both the control and experimental groups underwent posttest assessments for willingness to communicate and learner engagement. These posttest measures mirrored the pretest evaluations, allowing for comparing potential changes resulting from the intervention.

Sixth, to gain a deeper understanding of participants' experiences, a subset of 20 participants from the experimental group was selected for in-depth interviews. These semi-structured interviews provided an avenue for participants to elaborate on their perceptions of the impact of digital communication activities on their willingness to communicate and engagement levels. Seventh, the study employed a comprehensive data analysis approach encompassing quantitative and qualitative methods. The quantitative analysis involved comparing pretest and posttest scores within each group to identify significant changes in WTC and engagement levels. Concurrently, qualitative study was conducted on interview transcripts to uncover recurring themes and patterns in participants' narratives. Eighth, the findings from both quantitative and qualitative analyses were synthesized to offer a holistic comprehension of the influence of digital communication activities on EFL learners' willingness to communicate and engage. This synthesis uncovered how such activities shaped participants' attitudes, perceptions, and language learning experiences.

### 4.5 Data analysis

A rigorous statistical approach was adopted for the quantitative data analysis to derive meaningful insights from the participants' responses. The collected data, comprising pretest and posttest scores for the control and experimental groups, were subjected to statistical tests. First, descriptive statistics such as means, standard deviations, and frequency distributions were computed to provide an overview of participants' initial willingness to communicate and engage and any changes after the intervention. Next, inferential statistical methods were employed to determine the significance of the observed differences. Univariate analysis of variances (ANOVA) tests were used for comparing the groups' scores on types of engagement and types of WTC on pretests and posttests. In addition, the Levenw's test was used for estimating the homogeneity of the groups' variances.

Qualitative data analysis followed a systematic process of thematic analysis to extract meaningful patterns and themes from the transcribed interview data. Initially, the interview transcripts were carefully read and re-read to gain a comprehensive understanding of participants' narratives. Then, open coding was applied, involving the generation of initial codes that captured key ideas and concepts from the data. Subsequently, these initial codes were organized into potential themes. This process involved categorizing similar codes and identifying overarching patterns from participants' responses. Themes were refined through iterative review and researcher discussion to ensure accuracy and validity. Once the pieces were established, connections and relationships between them were explored to create a coherent narrative that captured participants' perceptions of the impact of digital communication activities on their willingness to communicate and engage. These themes were supported by relevant excerpts from the interview transcripts, lending credibility to the findings. Lastly, the qualitative analysis converged with the quantitative results to comprehensively understand the research phenomenon. This integration allowed for a rich interpretation of the data, enhancing the study's overall depth and validity.

### 4.6 Ethical considerations

This study was approved by the Institutional Review Board (IRB) of Shanxi Finance and Taxation College, Taiyuan, China. The IRB issued a letter (Number2023.1782) indicating that the study has no side effects on the participants of the research and it does not violate ethical considerations. Also, the informants willingly agreed to participate in the study and signed the informed consent form.

## 5 Results

### 5.1 Digital communication effects on the learners' engagement

The groups' scores on learner engagement and its components before and after the treatment were submitted to independent samples-t-tests. First, the results of t-tests for groups’ scores on learner engagement before the treatment are presented in Table 1..

**Table 1:** Means and Standard deviations of the groups’ scores on engagement.

Groups	Engagement	Mean	SD	N
Control	Affective	2.80	.73	40
	Cognitive	3.00	.78	40
	Behavioral	2.80	.68	40
	Total	2.90	.68	40
experimental	Affective	2.72	.70	40
	Cognitive	2.90	.53	40
	Behavioral	2.80	.60	40
	Total	2.88	.57	40

Control group's mean score on affective engagement was 2.80 (SD = 0.73), but the experimental group's mean score was 2.72 (SD = 0.78). Moreover, the control group's score on cognitive engagement was 3.00 (SD = 0.78), but the experimental group's mean score was 2.90 (SD = 0.53). In terms of behavioral engagement, both the control and experimental groups had mean scores of 2.80, with standard deviations of 0.68 and 0.60, respectively. For total engagement, the control group's mean score was 2.90 (SD = 0.68), whereas the experimental group's mean score was 2.88 (SD = 0.57). Results of ANOVA test as shown in Table 2, revealed that the difference between control and experimental groups' scores on all dimensions of engagement wad not statistically significant.

**Table 2:** ANOVA for the groups’ scores on engagement before the treatment.

Source	Type III Sum of Squares	df	Mean Square	F	Sig.
Corrected Model	2.362^a^	7	.337	.839	.555
Intercept	2578.5	1	2578.5	6415.7	.000
groups	1.089	1	1.089	2.710	.101
engagement	.886	3	.295	.735	.532
groups * engagement	.381	3	.127	.316	.814
Error	125.394	312	.402		
Total	2711.778	320			
Corrected Total	127.756	319			

a. R Squared = .018 (Adjusted R Squared = −.004).

As seen in Table 2, the model as a whole (Corrected Model) does not appear to be statistically significant (p = 0.555). Engagement and the interaction between Groups and Engagement do not seem to be statistically significant, as indicated by their higher p-values. The overall R Squared is 0.018, suggesting that the model explains a small proportion of the variance in the dependent variable. The adjusted R Squared is negative, indicating that the model may not be a good fit, suggesting that the groups were homogenous regarding learner engagement and its components. Results of the post-test are presented in Table 3 and Table 4.

**Table 3:** Means and Standard deviations of the groups’ scores on engagement.

Groups	engagement	Mean	SD	N
Control	Affective	2.9	.697	40
	Cognitive	2.9	.649	40
	Behavioral	2.80	.58	40
	Total	2.88	.605	40
experimental	Affective	3.67	.850	40
	Cognitive	3.61	.971	40
	Behavioral	4.01	.651	40
	Total	3.78	.802	40

Control group's mean score on affective engagement was 2.90 (SD = 0.69), but the experimental group's mean score was 3.67 (SD = 0.85). Moreover, the control group's score on cognitive engagement was 2.90 (SD = 0.85), but the experimental group's mean score was 3.61 (SD = 0.97). In terms of behavioral engagement, both the control (M = 2.80, SD = 0.58) and experimental groups (M = 4.01, SD = 0.65) had different mean scores. For total engagement, the control group's mean score was 2.88 (SD = 0.60), whereas the experimental group's mean score was 3.78 (SD = 0.80). Results of ANOVA test as shown in Table 4, revealed that the difference between control and experimental groups' scores on engagement wad statistically significant.

**Table 4:** ANOVA for the groups’ scores on engagement after the treatment.

Source	Type III Sum of Squares	df	Mean Square	F	Sig.	Partial Eta Squared
Corrected Model	69.153a	7	9.879	19.58	.001	.305
Intercept	3538.519	1	3538.519	7016.026	.001	.957
groups	64.080	1	64.080	127.05	.001	.53
engagement	.622	3	.207	.411	.745	.004
groups * engagement	4.033	3	1.344	2.665	.06	.025
Error	157.357	312	.504			
Total	3811.615	320				
Corrected Total	226.510	319				

a. R Squared = .305 (Adjusted R Squared = .290)

As seen in Table 4, the Corrected Model was highly significant (F(7, 312) = 19.58, p < 0.001, η^2^ = 0.305), indicating that the model as a whole effectively explains a significant proportion of the variance in the dependent variable. The intercept and groups both showed highly significant effects (F (1, 312) = 7016.026, p < 0.001, η^2^ = 0.957; F(1, 312) = 127.05, p < 0.001, η^2^ = 0.289, respectively), suggesting substantial contributions to the model. However, the main effect of engagement was not statistically significant (F (3, 312) = 0.411, p = 0.745, η^2^ = 0.004).

The proportion of variance explained by the model (R Squared) was substantial at 30.5 %, and the adjusted R Squared was 29 %, suggesting that the model remains robust when accounting for the number of predictors.

Research Question 2 Digital communication effects on the learners' willingness to communicate

The groups' scores on willingness to communicate and its components before and after the treatment were submitted to separate ANOVA tests. First, the results of t-tests for groups’ scores on WTC before the treatment are presented in Table 5.

**Table 5:** T-test for comparing groups’ score willingness to communicate before the treatment.

groups	WTC	Mean	SD	N
control	Speaking in class	23.74	1.98	40
	Reading in class	18.45	1.69	40
	Writing in class	21.50	2.68	40
	Comprehension	11.95	1.33	40
	Willingness to communicate (Total)	88.32	8.23	40
experimental	Speaking in class	23.18	2.073	40
	Reading in class	18.00	2.17	40
	Writing in class	21.20	2.92	40
	Comprehension	14.10	14.00	40
	Willingness to communicate (Total)	86.92	9.118	40

As shown in Table 5, the control group mean score for speaking in class was 23.74 (SD = 1.98), for reading in class was 18.45 (SD = 1.69), for writing in class was 21.50 (SD = 2.68), for comprehension was 11.95 (SD = 1.33), and the total willingness to communicate was 88.32 (SD = 8.23). Moreover, the experimental group mean score for speaking in class was 23.18 (SD = 2.073), for reading in class was 18.00 (SD = 2.17), for writing in class was 21.20 (SD = 2.92), for comprehension was 14.10 (SD = 14.00), and the total willingness to communicate was 86.92 (SD = 9.118). Results of ANOVA test verified that the groups’ mean scores on WTC and its components were not statistically significant. Results are shown in Table 6.

**Table 6:** ANOVA for the groups’ scores on willingness to communicate before the treatment.

Source	Type III Sum of Squares	df	Mean Square	F	Sig.	Partial Eta Squared
Corrected Model	305582.34	9	33953.593	892.351	.000	.954
Intercept	427768.11	1	427768.11	11242.3	.000	.966
groups	1.286	1	1.286	.034	.854	.000
WTC	305438.28	4	76359.572	2006.84	.000	.954
groups * WTC	142.731	4	35.683	.938	.442	.010
Error	14839.337	390	38.050			
Total	751217.00	400				
Corrected Total	320421.67	399

As shown in Table 6, the difference between the groups' willingness to communicate at the onset of the treatment was not statistically significant (F(1) = 0.034, p = 0.854 > 0.05). The interaction between groups and types of WTC was not significant, either (F(4) = 2006, p = 0.442 > 0.05). To see whether the treatment affect the learners' WTC, the groups’ scores on posttest were submitted to ANOVA test. Results are presented in Table 7, Table 8.

**Table 7:** Groups’ scores on willingness to communicate after the treatment.

groups	WTC	Mean	SD	N
control	Speaking in class	24.6	3.3	40
	Reading in class	18.6	4.1	40
	Writing in class	24	2.2	40
	Comprehension	12.6	4.3	40
	Willingness to communicate (Total)	79.8	13.9	40
experimental	Speaking in class	31.3	3.6	40
	Reading in class	24.3	4.1	40
	Writing in class	26.5	2.3	40
	Comprehension	18.6	2.4	40
	Willingness to communicate (Total)	107.8	12.4	40

**Table 8:** ANOVA test for the groups’ WTC after the treatment.

Source	Type III Sum of Squares	df	Mean Square	F	Sig.	Partial Eta Squared
Corrected Model	352884.110^a^	9	39209.346	912.129	.001	.955
Intercept	530613.729	1	530613.729	12343.69	.001	.969
Groups	5366.825	1	5366.825	124.849	.001	.823
WTC	346395.676	4	86598.919	2014.554	.001	.954
groups * WTC	1078.831	4	269.708	6.274	.001	.060
Error	16764.788	390	42.987			
Total	904083.000	400				
Corrected Total	369648.897	399

As seen in Table 7, the comparison between willingness to speak of the control group (M = 24.6, SD = 3.3) and the experimental group (M = 31.3, SD = 3.6) revealed a statistically significant difference. Moreover, in willingness to speak, a significant difference emerged between the control group (M = 18.6, SD = 4.1) and the experimental group (M = 24.3, SD = 4.1). Regarding desire to write, the experimental group (M = 26.5, SD = 2.3) outperformed the control group (M = 24, SD = 2.2) with statistical significance. Moreover, a significant difference was observed in the comprehension aspect of WTC between the control group (M = 12.6, SD = 4.3) and the experimental group (M = 18.6, SD = 2.4). Finally, regarding willingness to communicate as the primary variable, the experimental group (M = 107.8, SD = 12.4) displayed significantly higher scores than the control group (M = 79.8, SD = 13.9). Results of ANOVA test also (Table 8) verified that the difference between the control and experimental groups’ scores on WTC was statistically significant.

As seen in Table 8, the analysis of variance (ANOVA) yielded statistically significant results (F(9, 390) = 912.129, p < 0.001, η^2^ = 0.955), indicating that the Corrected Model effectively explained a substantial proportion of the variance in the dependent variable. The main effects for groups (F(1, 390) = 124.849, p < 0.001, η^2^ = 0.242) and WTC (Willingness to Communicate) (F(4, 390) = 2014.554, p < 0.001, η^2^ = 0.954) were both significant, highlighting the independent contributions of group differences and willingness to communicate to the observed variance. The interaction effect between groups and WTC was also significant (F(4, 390) = 6.274, p = 0.001, η^2^ = 0.060), suggesting a combined influence. The effect sizes, as indicated by partial eta squared values, were substantial for all factors, with the Corrected Model explaining 95.5 % of the variance. These findings underscore the importance of both individual and group factors in language learning outcomes, with a notable emphasis on willingness to communicate. Therefore, it can be strongly argued that the treatment.

Research Question 3 Language learners' perceptions of the use of digital communication activities

A qualitative case study methodology was employed to address the research question, entailing in-depth interviews with 20 language learners. This approach facilitated an intricate exploration of the advantages of digital communication activities in their language-learning journeys. Through meticulous analysis, ten prominent themes emerged, encapsulating the essence of the participants' experiences. The first theme, “*Enhanced Accessibility to Native Speakers*,” resonated deeply with 90 % of the language learners. This theme highlighted the remarkable opportunity digital communication provided in connecting learners with native speakers worldwide. Interviewee 1 substantiated this theme: “Engaging in online language exchange platforms has been invaluable in practicing with actual native speakers."

The second theme, aptly named “*Diverse Learning Resource*s,” illuminated the extensive array of online tools that learners could leverage. From language learning apps to interactive websites, participants highlighted how these resources enriched their vocabulary and grammar skills. Interviewee 3 remarked, “Having access to diverse resources online has really transformed the way I learn.” The third theme, “*Flexibility in Learning*,” underscored the advantage of digital communication for accommodating busy schedules. Learners could engage in language exchange or practice conversations at their convenience. Interviewee 5 affirmed, “Being able to practice speaking with someone across the globe, regardless of time zones, has been a game-changer."

The fourth theme, “*Real-World Contextualization*,” emphasized how digital communication facilitated learning within authentic contexts. Participants could engage in meaningful conversations about current events or personal experiences, aiding comprehension and retention. Interviewee 8 noted, “Talking about real-world topics online helped me understand how the language is used naturally."

The fifth theme, “*Instant Feedback and Correction*,” highlighted how digital communication enabled immediate feedback from peers or native speakers. This aspect was particularly beneficial for refining pronunciation and grammar skills. Interviewee 10 shared, “When I make a mistake, my language exchange partner corrects me right away, which helps me learn faster."

The sixth theme, *“Cultural Exchange,*” unveiled how digital communication activities transcended linguistic learning by fostering cross-cultural understanding. Participants found themselves immersed in the language and the cultural contexts of their conversation partners. Interviewee 12 reflected, “Through video calls, I learned the language and got insights into their way of life."

The seventh theme*, “Overcoming Communication Apprehension,”* addressed how digital communication activities provided a more comfortable environment for hesitant learners. The physical separation from face-to-face interactions alleviated anxiety and boosted confidence. Interviewee 15 stated, “Online interactions helped me overcome my fear of making mistakes in front of others."

The eighth theme, *“Varied Communication Modes,”* spotlighted the assortment of communication platforms available, such as text chats, voice calls, and video conferencing. This diversity enabled learners to practice and refine different language skills. Interviewee 18 mentioned, “I can practice writing in text chats and speaking in video calls, which covers a wide range of language skills."

The ninth theme, *“Self-Paced Progress Tracking,*” demonstrated how digital communication activities allowed learners to track their progress over time. Participants could revisit previous conversations and note their improvement, boosting motivation. Interviewee 20 acknowledged, “Scrolling back to my first conversations showed me how far I've come, which motivated me to keep going."

Lastly, the tenth theme, *“Global Networking,”* showcased how digital communication transcended geographical boundaries, fostering connections with fellow language enthusiasts worldwide. Participants could build a supportive community to share experiences and advice. Interviewee 20 shared, “I've made friends from different corners of the world, all brought together by our passion for language.” These ten themes collectively unveil the multifaceted advantages of digital communication activities in the language learning process, underscoring their pivotal role in shaping learners' experiences and proficiency.

## 6 Discussion

The findings of this study provide compelling evidence of the positive impact of digital communication activities on EFL learners' engagement across multiple dimensions. The comparison of pretest and posttest scores between the experimental and control groups revealed significant improvements in affective, cognitive, and behavioral engagement, as well as overall learner engagement. Regarding affective engagement, the experimental group displayed significantly higher levels than the control group. This aligns with prior research that highlights the role of digital communication activities in fostering a more emotionally connected and invested learning environment [19,20]. The increase in affective engagement suggests that these activities contribute to creating a more enjoyable and meaningful learning experience, likely due to digital platforms' collaborative and interactive nature [69].

Cognitive engagement also exhibited substantial improvement in the experimental group. This finding is consistent with the Cognitive Theory of Learning, which underscores the importance of comprehension and understanding in learning [28]—engaging in digital communication exposed learners to diverse language resources and interactions, promoting more profound experience and knowledge acquisition [[29], [30], [31]]. The statistically significant increase in cognitive engagement underscores the efficacy of these activities in enhancing learners' grasp of language concepts.

The observed rise in behavioral engagement further reinforces the positive impact of digital communication activities. Learners in the experimental group demonstrated higher levels of active participation and involvement in learning tasks than their counterparts in the control group. This echoes the sociocultural theory's emphasis on the role of social interactions in shaping behavior and learning [20]. The collaborative nature of digital communication platforms encourages learners to actively contribute, share ideas, and collaborate, leading to enhanced behavioral engagement.

Moreover, the total learner engagement score in the experimental group significantly surpassed that of the control group. This holistic measure affirms the comprehensive impact of digital communication activities on learners' overall engagement. The findings align with previous research that highlights the potential of digital platforms in creating a more engaging and interactive learning environment [31,34].

The comparison of effect sizes further elucidates the magnitude of the intervention's impact. The moderate effect size underscores the robustness of the findings. This suggests that digital communication activities consistently enhance various dimensions of engagement, with particularly pronounced effects on cognitive and behavioral engagement.

The findings also illuminated the significant impact of digital communication activities on EFL learners' willingness to communicate (WTC) across different dimensions. The comparison of scores between the experimental and control groups underscores the positive effects of the intervention on various aspects of communication willingness. Willingness to communicate in speaking exhibited a remarkable increase in the experimental group, as evidenced by the significantly higher mean score than the control group. This finding aligns with studies emphasizing the potential of digital communication platforms to enhance speaking skills and encourage learners to engage in spoken interactions [40]. The large effect size (Eta = 0.954) suggests a substantial positive impact of the intervention, indicating that learners exposed to digital communication activities were notably more willing to engage in spoken and written communication.

Similarly, the experimental group's willingness to communicate in writing witnessed a significant improvement. The higher mean score in the experimental group compared to the control group underscores the efficacy of digital communication activities in fostering greater engagement in written communication tasks. This finding aligns with the cognitive theory of learning, highlighting the role of comprehension and understanding in language acquisition, particularly in writing [28].

Comprehension aspect of WTC also experienced a marked increase in the experimental group. The groups' statistically significant difference in comprehension engagement indicates the intervention's positive impact on learners' ability to understand and interpret language. This aligns with previous research findings that emphasize the role of digital communication activities in enhancing language comprehension [[35], [36], [37], [38], [39]]. The large effect size (0.954) underscores the substantial improvement in comprehension engagement resulting from the intervention.

Finally, as a comprehensive measure, the overall willingness to communicate exhibited a substantial positive effect due to the intervention. The experimental group displayed significantly higher willingness to communicate scores than the control group. This indicates that learners exposed to digital communication activities were more inclined to communicate across various modes, including speaking, writing, and comprehension. The considerable effect size [0.954) emphasizes the intervention's substantial impact on learners' willingness to communicate.

In conclusion, the findings of this study provide strong evidence of the positive impact of digital communication activities on EFL learners' willingness to communicate. The intervention significantly increased learners' willingness to engage in speaking, writing, comprehension activities, and overall communication. These findings highlight the potential of digital platforms in cultivating various aspects of communication willingness among language learners.

The qualitative case study approach facilitated an in-depth exploration of digital communication activities' benefits in language learning. Twenty language learners were interviewed, revealing ten prominent themes highlighting the advantages of these activities. The first theme, “Enhanced Accessibility to Native Speakers,” emphasized exposure to authentic language use [34,37]. "Diverse Learning Resources,” the second theme, aligned with cognitive theory [28]) and catered to varied learning preferences [20,37]. The third theme, “Flexibility in Learning,” echoed sociocultural theory [19], accommodating schedules and fostering empowerment [32]. "Real-world contextualization,” the fourth theme, immersed learners in meaningful contexts [34,35]. "Instant Feedback and Correction,” the fifth theme, aligned with cognitive theory [28], accelerating learning [20,33]. "Cultural Exchange,” the sixth theme, facilitated intercultural competence [1]. "Overcoming Communication Apprehension,” the seventh theme, eased anxiety [3]. "Varied Communication Modes,” the eighth theme, engaged learners diversely [35]. "Self-Paced Progress Tracking,” the ninth theme, fostered metacognition. Lastly, “Global Networking,” the tenth theme, created a learner community [69]. These themes substantiated the positive impact of digital platforms on language learning [[37], [38], [39], [40]].

The convergence of qualitative and quantitative findings in this study provides a rich and nuanced understanding of the multifaceted impact of digital communication activities on EFL learners. Quantitatively, the study revealed significant improvements in affective, cognitive, and behavioral engagement and overall learner engagement within the experimental group exposed to these activities. The qualitative case study, through in-depth interviews, unveiled ten prominent themes that underscored the diverse benefits of digital communication activities. Remarkably, these qualitative themes aligned with and provided context to the quantitative improvements observed in the experimental group. For instance, the theme “Enhanced Accessibility to Native Speakers” resonated with the significant increase in affective engagement, suggesting that exposure to authentic language use through digital platforms contributed to a more emotionally connected learning experience. Similarly, the theme “Diverse Learning Resources” aligned with the substantial improvement in cognitive engagement, reflecting how varied learning materials catered to different preferences and promoted deeper comprehension.

The qualitative findings' theme “Flexibility in Learning” mirrored the observed rise in behavioral engagement, emphasizing how the sociocultural aspect of flexibility accommodated individual schedules and empowered learners to participate actively. "Real-world Contextualization” and “Cultural Exchange” themes complemented the positive impact on learner engagement, emphasizing the meaningful contexts and intercultural competence fostered through digital communication activities. Furthermore, qualitative themes like “Overcoming Communication Apprehension” and “Varied Communication Modes” provided context to the significant increase in willingness to communicate in the experimental group, particularly in speaking and writing. The theme “Global Networking” mirrored the broader enhancement in overall willingness to communicate, emphasizing creating a learner community through digital platforms.

This alignment between qualitative themes and quantitative improvements strengthens the validity and depth of the study's findings. The positive effects observed in engagement and willingness to communicate are statistically significant and intricately tied to the qualitative experiences and perceptions of the learners engaged in digital communication activities. These findings provide a comprehensive picture of how such activities contribute to a more enriched and participatory language learning environment.

## 7 Conclusions

In conclusion, this study provides robust evidence of the positive impact of digital communication activities on EFL learners' engagement across multiple dimensions [70]. The experimental group exhibited significant affective, cognitive, and behavioral engagement improvements compared to the control group. This aligns with prior research, highlighting the role of digital activities in creating an emotionally connected learning environment. Additionally, cognitive engagement improved, substantiating the efficacy of these activities in enhancing learners' understanding and knowledge acquisition. The observed rise in behavioral engagement underscores the role of social interactions facilitated by digital platforms [71]. The study's findings indicate that digital communication activities hold the potential to foster comprehensive learner engagement. The comparison of effect sizes further confirms the intervention's effectiveness, consistently enhancing various dimensions of engagement. Notably, the intervention positively impacted EFL learners' willingness to communicate, particularly in speaking and writing. The substantial effect sizes underscore the significance of this impact.

In summary, this study supports the value of digital communication activities in promoting engagement and communication willingness among EFL learners. These findings encourage the integration of digital tools in language learning contexts. The qualitative insights from the case study further emphasize the benefits of these activities, highlighting themes such as accessibility to native speakers, diverse learning resources, flexibility, real-world contextualization, and more. Together, these results contribute to understanding how digital platforms can enhance language learning experiences.

While the study offers valuable insights, it is important to acknowledge certain limitations. The relatively small sample size may impact the findings' generalizability, and the intervention's short duration raises questions about its long-term sustainability. Uncontrolled contextual factors and external influences could affect the outcomes. Further research opportunities include exploring the effects of digital communication activities across diverse learner profiles, investigating the roles of teachers in facilitating such activities, and exploring the integration of these tools within curricula. Longitudinal studies could provide insights into the persistence of observed improvements over extended periods. Furthermore, comparative studies could help discern the varying impacts of different digital platforms on engagement and communication willingness.

## 8 Practical implications

The findings of this study provide a robust foundation for practical implications aimed at improving language learning programs, offering valuable insights for educators, policymakers, and program developers in the field of English as a Foreign Language (EFL). The integration of digital communication activities can be strategically informed in different ways. Firstly, the design of culturally rich activities is paramount. The positive impact of digital communication on intercultural competence underscores the importance of exposing learners to diverse cultural contexts. Incorporating collaborative projects, online discussions, and language exchange programs can provide authentic experiences, fostering a deeper understanding of language within cultural nuances. Secondly, recognizing the significance of diverse learning resources in promoting cognitive engagement, educators and policymakers should invest in creating or curating multimedia materials such as videos, podcasts, and interactive modules. This ensures a variety of resources to cater to different learning preferences and enhance overall comprehension.

Flexibility is identified as a key theme; therefore, policymakers should encourage the integration of flexible learning schedules. This can empower educators to utilize asynchronous communication tools, accommodating diverse learner schedules and fostering a sense of empowerment and control over the learning process. Thirdly, incorporating real-world contextualization into digital activities is crucial for maximizing engagement. Simulated real-life scenarios, virtual cultural experiences, and practical language use in digital platforms can immerse learners in meaningful contexts, making the language learning experience more relevant and applicable. Fourthly, Recognizing the importance of instant feedback, language learning platforms and educators should implement features that provide timely and constructive feedback. This addresses learner queries promptly and corrects language use effectively, accelerating the learning process. Fifthly, addressing communication apprehension is essential, and educators should leverage digital communication activities to create a supportive and encouraging environment. Activities that gradually expose learners to spoken and written interactions, coupled with supportive feedback, can contribute to overcoming communication apprehension. Sixthly, promoting varied communication modes aligns with the multifaceted improvement observed in willingness to communicate. Language programs should incorporate a balance of activities that encourage learners to express themselves in various ways, ensuring a holistic development of language skills.

Seventhly, supporting self-paced progress tracking is essential for learner autonomy. Platforms and programs should integrate features that enable learners to track their progress, fostering metacognition. This self-paced approach, coupled with digital tools that facilitate progress monitoring, allows learners to take control of their learning journey and reflect on their language development. Lastly, professional development for educators is critical. Policymakers and institutions should invest in professional development opportunities to enhance educators' digital literacy skills. Training programs can equip educators with the knowledge and skills needed to integrate digital communication activities into their teaching practices effectively.

## Ethical approval consent

The ethical approval committee of Shanxi Finance and Taxation College (No: 823/2023), indicating that the study was conducted in line with the guidelines and ethical considerations of the institute. All subjects gave their informed consent for inclusion before participating in the study.

## Data availability

The data would be available upon request.

## Funding

Wuhan Qingchuan University, Grant Number: JY202332.

## CRediT authorship contribution statement

**Ruyu Han:** Writing – review & editing, Writing – original draft, Project administration, Data curation, Conceptualization. **Goudarz Alibakhshi:** Writing – review & editing, Writing – original draft, Formal analysis, Data curation. **Lu Lu:** Writing – original draft, Methodology, Funding acquisition, Conceptualization. **Akram Labbafi:** Validation, Methodology, Investigation.

## Declaration of competing interest

The authors declare that they have no known competing financial interests or personal relationships that could have appeared to influence the work reported in this paper.
